# 11p Microdeletion including *WT1 *but not *PAX6*, presenting with cataract, mental retardation, genital abnormalities and seizures: a case report

**DOI:** 10.1186/1755-8166-2-6

**Published:** 2009-02-17

**Authors:** Gitte J Almind, Karen Brøndum-Nielsen, Regitze Bangsgaard, Peter Baekgaard, Karen Grønskov

**Affiliations:** 1 Kennedy Center, Glostrup, Denmark; 2 Department of Ophthalmology, Glostrup Hospital, Glostrup, Denmark; 3 Department of Paediatrics, Glostrup Hospital, Glostrup, Denmark

## Abstract

WAGR syndrome (Wilms' tumor, aniridia, genitourinary abnormalities and mental retardation) and Potocki-Shaffer syndrome are rare contiguous gene deletion syndromes caused by deletions of the 11p14-p12 chromosome region.

We present a patient with mental retardation, unilateral cataract, bilateral ptosis, genital abnormalities, seizures and a dysmorphic face. Cytogenetic analysis showed a deletion on 11p that was further characterized using FISH and MLPA analyses. The deletion (11p13-p12) located in the area between the deletions associated with the WAGR and Potocki-Shaffer syndromes had a maximum size of 8.5 Mb and encompasses 44 genes. Deletion of *WT1 *explains the genital abnormalities observed. As *PAX6 *was intact the cataract observed cannot be explained by a deletion of this gene. Seizures have been described in Potocki-Shaffer syndrome while mental retardation has been described in both WAGR and Potocki-Shaffer syndrome. Characterization of this patient contributes further to elucidate the function of the genes in the 11p14-p12 chromosome region.

## Background

The clinical association of Wilms' tumor, aniridia, genitourinary abnormalities and mental retardation (WAGR) is a contiguous gene deletion syndrome caused by a deletion on the short arm of chromosome 11. The syndrome is caused by haploinsufficiency for the *PAX6 *gene (causing aniridia) and the *WT1 *gene (predisposing Wilms' tumor, genital abnormalities and nephropathies). Aniridia is clinically required for the diagnosis [1]. Most WAGR patients are mentally retarded to some extent, and obesity has occasionally been noted, however the genetic causes for these traits have not been elucidated [2-5]. Recently Xu et al hypothesised that the *SLC1A2*, *PRRG4 *and *BDNF *genes might contribute to the abnormal mental development [6].

Potocki-Shaffer syndrome (PSS) is another gene deletion syndrome caused by a deletion on chromosome 11, but more proximal (11p11.2) than the WAGR deletion. The syndrome is characterized by foramina parietalia permanga, multiple exostoses and in some cases craniofacial dysostosis and mental retardation. Haploinsufficiency for *EXT2 *and *ALX4 *explains exostoses and parietal foramina respectively [7-9].

We present here a patient with an 8.5 Mb deletion on chromosome 11 located in the area between the WAGR and PSS deletions (11p13-p12).

## Case presentation

A 15-year-old boy was referred to us for cytogenetic studies. He was the first child of unrelated parents. Pregnancy and delivery at term (41 weeks of gestation, birth weight 3220 g and length 50 cm) were normal and uneventful.

**Figure 1:**
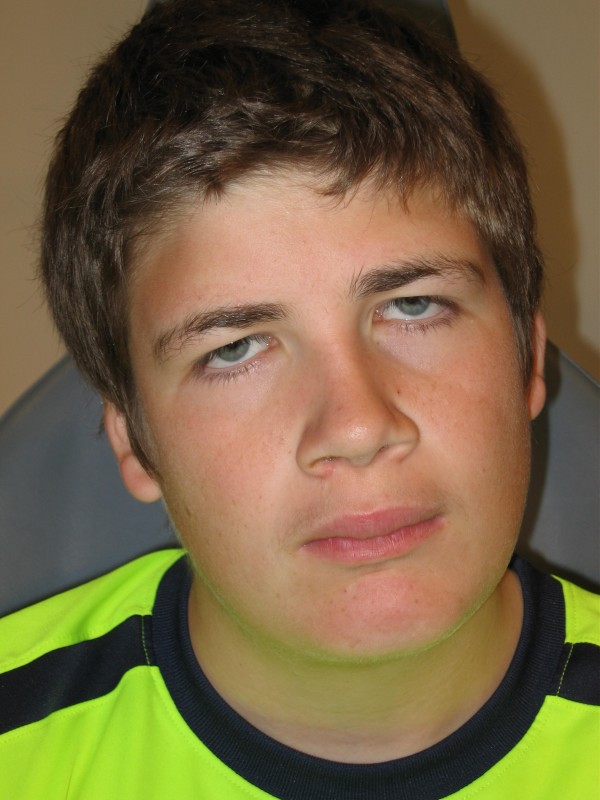
**Patient presenting with depressed nasal bridge, maxillary overbite and bilaterally down slanting eyes with ptosis**.

The patient is mildly to moderately mentally retarded and attends special school. His face is dysmorphic with a depressed nasal bridge, folded ears (especially on the right side), and a maxillary overbite and bilaterally down slanting eyes with ptosis (figure 1). Further ophthalmologic examination revealed unilateral cataract, astigmatism and myopia (right eye).

In the neonatal period cryptorchidism and hypospadias were noted. Upon surgery the testes were found atrophic as well. Nine years old he began to have seizures that were treated medically. After medication a weight gain from the 75 centile to just beneath the 97 centile (weight-for-height ratio) was noted.

**Figure 2:**
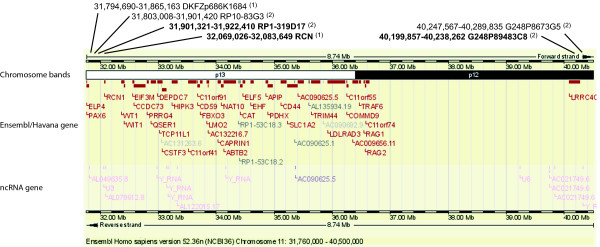
**Ensemble map of chromosome 11 (31,760,000–40,289,835)**. Above map are shown localization of probes used in FISH and MLPA analyses. Probes deleted on the derivative chromosome 11 are shown in bold. (1) MLPA probe, (2) FISH probe.

Cytogenetic analyses were performed after obtaining informed consent. Conventional cytogenetic preparations were made from PHA-stimulated peripheral blood. At first a normal male karyotype 46,XY was found, but upon re-examination using R-banding with a quality corresponding to approximately 550 bands an 11p deletion was revealed with some uncertainty. Comparative genomic hybridization (chromosome CGH) showed a 11p13 deletion. Fluorescence in situ hybridization (FISH) analysis was carried out using BACs and fosmid clones (CHORI BACPAC resource ). The positions of relevant probes are shown in figure 2. The clones for FISH analysis were labeled with biotin using a nick-translation kit following the manufacturer's protocol (Roche Molecular Biochemicals). The probes were preannealed with Cot1 DNA in hybridization mix, denatured for 5 minutes at 75°C, and added to the denatured chromosomal slides. Hybridization was carried out overnight. Signals were detected using 2–3 rounds of amplification with FITC (fluoresceinisothiocyanate) conjugated avidin and anti-avidin antibodies. The chromosomal slides were counterstained with propidium iodide and DAPI. The chromosomes were viewed using Leica FISH station Q550CW using the DMRXA microscope equipped with appropriate filters. A minimum of 20 metaphases was analyzed. MLPA analysis was performed using the P219 kit from mrc-Holland following manufacturer's instructions. The positions of relevant MLPA probes are shown in figure 2.

**Figure 3:**
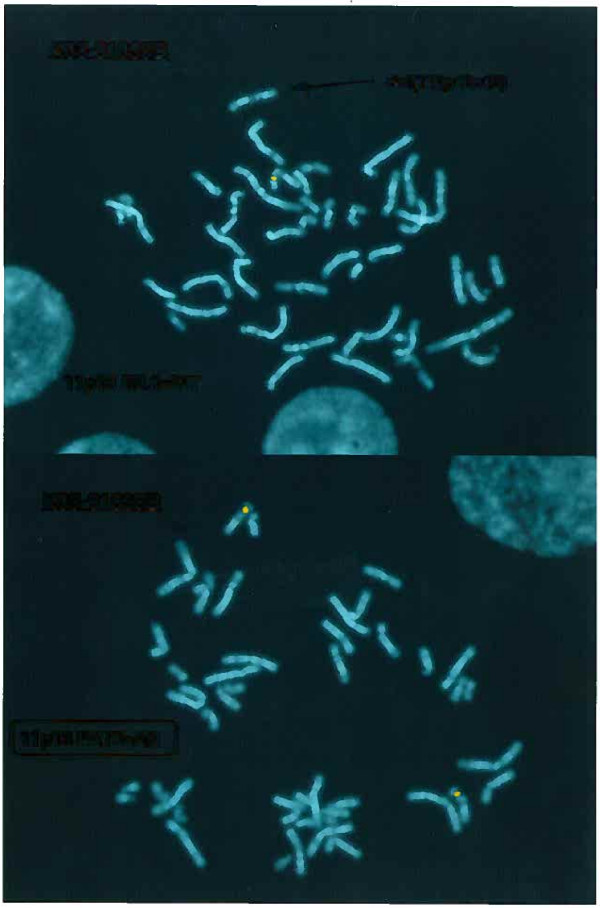
**a) FISH analysis using probe B2.1 (WT1 gene) showing signal from only one chromosome 11**. b) FISH analysis using FAT5 probe (PAX6 gene) showing signals from both chromosomes 11.

FISH analysis using probe B2.1 (*WT1*, 11p14.1 [10]) showed deletion of Wilms' tumor-locus while FISH analysis using probe FAT5 (aniridia-locus, *PAX6 *gene, 11p14.1 [10]) showed signals from both chromosomes 11 (figure 3). A further mapping of the deletion with FISH analysis using BAC and fosmid clones revealed a deletion of approximately 8.5 Mb. This is the maximum size as it is measured from the distal point of the probe juxtaposed to the distal probe deleted (i.e. BAC clone RP10-83G3 juxtaposed to deleted BAC clone RP1-319D17) to the proximal point of the probe juxtaposed to the proximal probe deleted (i.e. fosmid clone G248P8673G5 juxtaposed to deleted fosmid clone G248P89483C8). Thus the distal breakpoint mapped between position 31,803,008 (BAC clone RP10-83G3 not deleted) and position 31,922,410 (BAC clone RP1-319D17 deleted), while the proximal breakpoint mapped between position 40,199,857 (fosmid clone G248P89483C8 deleted) and position 40,289,835 (fosmid clone G248P8673G5 not deleted) (figure 2).

## Conclusion

The patient presented in this work shows mental retardation, unilateral cataract, bilateral ptosis, genital abnormalities, seizures and a dysmorphic face. It is uncertain whether the obesity observed is a side effect of the treatment for seizures or if it is part of the syndrome. The deletion breakpoints were mapped using FISH and MLPA analyses. The distal breakpoint was mapped between the *PAX6 *and *RCN1 *genes, while the proximal breakpoint was mapped to lie either within or proximal to the *LRRC4C *gene (Figure 2). The deletion encompasses a total of 44 genes or open reading frames, including the *WT1 *gene, which explains the genital abnormalities observed. As *PAX6 *is left intact the cataract observed cannot be explained by a deletion of this gene. One explanation may be that regulatory elements of *PAX6 *is deleted as such elements have been demonstrated [11]. Regulatory elements, located 5' to the *Pax6 *gene important for lens induction have been identified in mouse [12,13]. These upstream elements were not involved in the deletion in the patient described here, and it is purely speculative that others could exist. Another explanation is that the cataract is due to other genes involved in the deletion or causes unrelated to the deletion.

McGaughran et al. reported a case with a cytogenetic visible 11p deletion (del11(p11.2p14) [14]. This patient exhibited features of both WAGR as well as PSS; developmental delay but no seizures. Bremond-Gignac et al described a further case with 11p deletion encompassing *EXT2*, *ALX4*, *WT1 *and *PAX6 *genes showing features of both WAGR and PSS [15]. In addition, the patient showed obesity. Recently Xu et al. reported 31 WAGR cases and identified the genes deleted in each case using oligonucleotide array-CGH [6]. Three of these cases had seizures. One patient had an intact *PAX6 *gene, however, ophthalmologic findings of the patient were not described since the scope of the paper was the mental retardation and autism observed in WAGR patients. Xu et al hypothesizes that *SLC1A2 *and *BDNF *contributes to the autism and mental retardation [6]. In the patient presented here the *SLC1A2 *gene was deleted however the *BDNF *gene was intact.

Seizures have been described in PSS while mental retardation has been described in both WAGR and PSS. There are no obvious candidate genes for these symptoms, but our patient may help to further delineate phenotype-genotype relations in the area.

## Abbreviations

WAGR: Wilms' tumor, Aniridia, Genitourinary abnormalities and mental Retardation; PSS: Potocki-Shaffer Syndrome; FISH: Fluorescence In Situ Hybridization; MLPA: Multiplex Ligation Dependent Probe Amplification; CGH: Comparative Genomic Hybridization

## Consent

Written informed consent was obtained from the parents of the patient for publication with any accompanying images.

## Competing interests

The authors declare that they have no competing interests.

## Authors' contributions

GJA reviewed all clinical data and prepared the manuscript. PB and RB contributed clinical information. KG and KBN provided valuable support and supervised the practical work in the laboratory. All authors read and approved final manuscript.
